# Machine learning assisted plasmonic metascreen for enhanced broadband absorption in ultra-thin silicon films

**DOI:** 10.1038/s41377-024-01723-8

**Published:** 2025-01-09

**Authors:** Waqas W. Ahmed, Haicheng Cao, Changqing Xu, Mohamed Farhat, Muhammad Amin, Xiaohang Li, Xiangliang Zhang, Ying Wu

**Affiliations:** 1 Division of Computer, Electrical and Mathematical Sciences and Engineering, King Abdullah University of Science and Technology (KAUST), Thuwal, 23955-6900 Saudi Arabia; 2 College of Engineering, Taibah University, Madinah, 42353 Saudi Arabia; 3 Division of Physical Science and Engineering, King Abdullah University of Science and Technology (KAUST), Thuwal, 23955-6900 Saudi Arabia; 4 Department of Computer Science and Engineering, University of Notre Dame, Notre Dame, IN 46556 USA

**Keywords:** Photonic devices, Metamaterials

## Abstract

We propose and demonstrate a data-driven plasmonic metascreen that efficiently absorbs incident light over a wide spectral range in an ultra-thin silicon film. By embedding a double-nanoring silver array within a 20 nm ultrathin amorphous silicon (a-Si) layer, we achieve a significant enhancement of light absorption. This enhancement arises from the interaction between the resonant cavity modes and localized plasmonic modes, requiring precise tuning of plasmon resonances to match the absorption region of the silicon active layer. To facilitate the device design and improve light absorption without increasing the thickness of the active layer, we develop a deep learning framework, which learns to map from the absorption spectra to the design space. This inverse design strategy helps to tune the absorption for selective spectral functionalities. Our optimized design surpasses the bare silicon planar device, exhibiting a remarkable enhancement of over 100%. Experimental validation confirms the broadband enhancement of light absorption in the proposed configuration. The proposed metascreen absorber holds great potential for light harvesting applications and may be leveraged to improve the light conversion efficiency of ultra-thin silicon solar cells, photodetectors, and optical filters.

## Abstract (web summary)

Silver nanoring metastructures designed via AI dramatically boost light absorption in ultra-thin silicon films, achieving high efficiency for solar and photonic applications.

## Graphical abstract

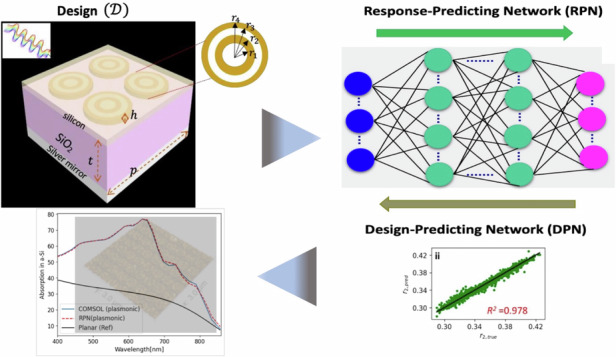

## Introduction

Modern optoelectronic devices need precise control of the absorption spectrum, including intensity, bandwidth, and spectral selectivity. Numerous approaches have been put forward to engineer the absorption properties, such as metamaterials^1^, nanostructured films^2^, plasmonic nanoantennas^3^, ultrathin semiconductors^4^, coherent perfect absorbers^5^, and time-reversed lasers^6^. While these mechanisms offer strong absorption, they typically operate within a narrow bandwidth. Optical devices, such as photodetectors, solar cells, and optical filters, are facing additional challenges, where broadband absorption with maximal efficiency is highly desired with *thin* active layers to expand their functionalities. Since light absorption depends on the conversion of photon energy into electrical energy in such devices^7^, the photon absorption is improved by increasing the thickness of the active material. Yet, as the thickness of the active layer increases to enhance recombination, the absorbed energy being captured as electrical energy at the electrodes is likely to decrease due to inherent intrinsic losses^8^. To overcome this trade-off, it becomes crucial to increase the total path length of light within the active layer without physically thickening it. Light trapping schemes have been developed to address this fundamental trade-off^9,10^, enabling enhanced optical absorption of ultra-thin active layers across a wide spectral range.

To achieve coherent light trapping, various techniques have been employed in recent years. When the active layer thickness exceeds the wavelength^11^, surface texturing and randomization of the photon density-of-states are commonly used to enhance absorption. In the case of thin active layers, subwavelength grating structures are employed to redistribute the photon density-of-states from selective spectral regions with enhanced absorption. Another light-trapping strategy involves leveraging surface-plasmon polariton (SPP) resonances, excited by metallic nanoparticles and/or periodic nanopatterned metallic films^12^, allowing a substantial improvement of light localization^13^ and hence absorption^14,15^. A semiconductor material embedded within metallic nanoparticles acts as an antenna for incident light and stores energy in the localized surface-plasmon resonance of the nanoparticles, which results in strong optical scattering as well as strong near-field enhancement^16^.

Metamaterials have emerged as a promising candidate for perfect absorption across different wavelength regimes^17–19^. By combining resonators of different frequencies, such as distributing them over a period^20,21^ or stacking metal–dielectric layers together^22,23^, the absorption spectrum can be broadened. Metamaterial absorbers generally rely on thin absorbing nanostructure surfaces; a dielectric spacer sandwiched between a metal mirror (ground plane) and a structured top layer. The metallic structures on the thin absorbing metasurface stimulate resonant currents that couple to the metallic mirror, generating high-intensity fields between the metal mirror and the dielectric spacer. A near-perfect absorption may be achieved with different configurations of the meta-absorber that lends itself to many practical applications, including energy harvesting^24^, imaging^25^, emitting^26^, sensing^27^, and many others^28,29^.

In this study, we propose a plasmonic meta-absorber that improves absorption in the visible range by enabling SPP resonances to be matched within the intrinsic absorption spectrum of silicon, thus improving absorption. Through careful engineering of plasmonic nanostructures, we achieve light localization and folding into a thin silicon film. Traditional approaches for determining the design parameters rely on conventional optimization methods^30–32^ which are computationally intensive and have limitations in terms of accuracy and practicality. In addition, inverse design requires many cycles of modeling and iterative searching in a large design space to predict the desired optical response, making it even more challenging.

**Fig. 1:**
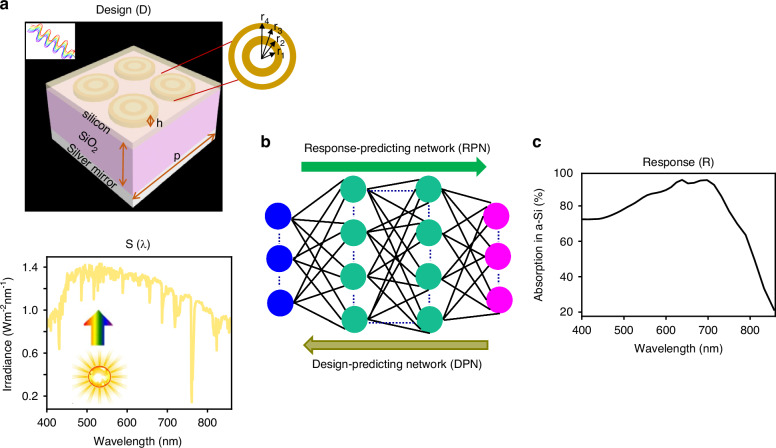
Machine learning-assisted meta-absorber for enhanced broadband absorption. **a** Schematic of the structure of the proposed meta-absorber consisting of four concentric silver nanorings embedded in an a-Si active layer (thickness 20 nm) on the top, a dielectric spacer SiO_2_ layer in the middle, and a silver mirror acts as a back reflector at the bottom. The structure is illuminated by incoming light covering the spectrally dense region of the solar spectrum *S*(*λ*), as shown in the lower panel. The inset on the top right depicts the front view of the silver nanorings pattern. **b** The proposed RPN can predict the absorption spectra from the parameters of the meta-absorber. In turn, the inverse DPN can predict the design parameters of the meta-absorber for a given absorption spectra. Blue and pink circles represent design and response spaces, respectively. **c** Broadband absorption spectrum of the 20 nm silicon layer of the meta-absorber

Recently, data-driven approaches have shown promising results in solving non-intuitive problems requiring optimization through their excellent generalizability^33,34^. Such methods can automatically produce the design parameters of any target within a few seconds without trial-and-error and are, therefore, simpler and more efficient than conventional methods^35,36^. The potential of AI-based techniques in solving physical problems has been demonstrated in a number of early studies^37–43^. In particular, for photonic design, various deep-learning approaches^44–52^, employing tandem training of deterministic and complex generative models for probabilistic representation, have significantly pushed the field forward. In this work, we develop a deep learning framework for automatic modeling and optimization of three-dimensional (3D) meta-absorbers, as shown in Fig. 1. This deep learning framework consists of bidirectional neural networks aimed at solving both the forward and inverse problems. In the forward path, a response predicting network (RPN), a fast-prototyping tool, is employed to predict the full optical response corresponding to the geometry of the meta-absorber. In the reverse path, a design predicting network (DPN) is leveraged to predict the full optical response (see Fig. 1). The proposed model learns the hidden trends between the design space and the absorption spectrum through supervised learning for tailoring the spectral characteristics to fulfill the requirements of a device for solar frequency window applications. It offers a straightforward solution to the inverse problem using a single neural network. The enhanced absorption in the proposed metascreen has been demonstrated both theoretically and experimentally. The photocurrent (short-circuit current) density is significantly improved, i.e., up to 100–180% increase with the optimized design parameters, as compared to planar devices.

## Results

Figure 1 schematically depicts the 3D structure of the proposed metascreen absorber, consisting of a top metasurface, a dielectric spacer, and a bottom metallic mirror. The metasurface comprises periodically arranged double concentric silver nanorings (shown in the inset of Fig. 1a), which are embedded in an ultra-thin a-Si layer with a thickness of 20 nm. The nano-shells contain alternating dielectric (silica) and metallic (silver) layers, whose geometric parameters are the radii *r*_1_–*r*_4_ and thickness, *h*, as shown in the inset of Fig. 1a. The dielectric spacer layer, made of SiO_2_ with thickness *t*, plays a crucial role to control the electromagnetic field interference between the top metasurface and the bottom metallic mirror. Meta-absorbers are characterized by their reflective properties because the bottom metallic mirror eliminates the transmission channel. The period of a single unit-cell is 400 nm. The device is illuminated by linearly polarized light with a normal incident angle. It is expected that the a-Si active layer will rapidly absorb light through near-field concentration capability of excited SPP modes in the nanorings. The light absorption capability of the a-Si is quantified from the ratio of spectral power absorbed inside the a-Si and the incident power from the source, and is defined as: $A=\frac{\omega {\varepsilon }_{0}\iiint {\rm{Im}}[\varepsilon (\omega )]{\left|E(x,y,z)\right|}^{2}{{\rm {d}}x{\rm {d}}y{\rm {d}}z}}{{P}_{{{\rm {in}}}}}$ where *P*_in_ is the projected incident power, *E* is the electric field within the active region, *ω* is the angular frequency of the incident light, Im[*ε*(*ω*)] is the imaginary part of the complex relative permittivity of the a-Si, and *ε*_0_ is the permittivity of free space.

To train our deep learning network, we use COMSOL Multiphysics^53^ to generate a large set of synthetic data from 3D simulations of the device structure. The generated data set contains 11,000 samples, where each sample is composed of design parameters of plasmonic meta-absorber and the corresponding absorption spectrum for the normally incident field. While material properties in each layer of the meta-absorber are fixed, the design space contains six geometrical parameters characterizing the plasmonic metascreen, i.e., *r*_1_–*r*_4_, *h* and the spacer layer thickness, *t*. The upper and lower bounds of the design space are 30 nm ≤ *r*_1_ ≤ 50 nm, 70 nm ≤ *r*_2_ ≤ 90 nm, 110 nm ≤ *r*_3_ ≤ 130 nm, 150 nm ≤ *r*_4_ ≤ 170 nm, 8 nm ≤ *h* ≤ 16 nm and 30 nm ≤ *t* ≤ 70 nm, for data generation. We compute the discretized absorption spectra *R* = [*A*_1_, *A*_2_, *A*_3_, *A*_4_... ... ...*A*_41_] for the random design space ${\mathscr{D}}$ = [*r*_1_, *r*_2_, *r*_3_, *r*_4_, *h*, *t*] in the wavelength range 400 nm ≤ *λ* ≤ 860 nm. We randomly divide the generated data into three groups, which contain 8000 samples for training, 1500 samples for validation, and the rest 1500 samples for final testing.

Following this step, we train the two neural networks (RPN and DPN) to learn the multivariate relationship between the geometric parameters and spectral characteristics. In the RPN, the input to the neural network is the design space (a 1 × 6-dimensional vector of [*r*_1_, *r*_2_, *r*_3_, *r*_4_, *h, t*]), and the output is the absorption spectrum (a 1 × 41-dimensional vector). Inversely, the DPN has the absorption spectrum as input, and the design space as prediction output. The neural network outputs are compared with numerical simulation results^53^ for calculating the loss function values, and the network weights are optimized to minimize the differences measured by the loss function. To stabilize the training process for minimizing the loss function, we apply normalization on the design parameters ${\mathscr{D}}$. Specifically, the design parameters are scaled by ${\mathscr{D}}/{{\mathscr{\parallel{D}\parallel}}}_{2}$, where ${{||}{\mathscr{D}}{||}}_{2}=\sqrt{{\sum }_{i}{{\mathscr{D}}}_{i}^{2}}$. This normalization will ensure that the six parameters in ${\mathscr{D}}$ remain within a standardized range and maintain their relative proportions. In this way, the gradients involved in the backpropagation process are less likely to become excessively large or small, promoting a smoother and more consistent neural network training process. The validation data set is used to avoid the overfiting during the training process. We evaluate the performance of the trained neural networks by applying them to the testing samples, which are unseen during training to show the ability to accurately predict the fabricated nanostructure’s parameters beyond simulations. The training of these networks and the optimal enhanced broadband absorption are discussed in the next sections, followed by experimental verification of the proposed meta-absorber for a selective spectral response.

### Response predicting network

**Fig. 2:**
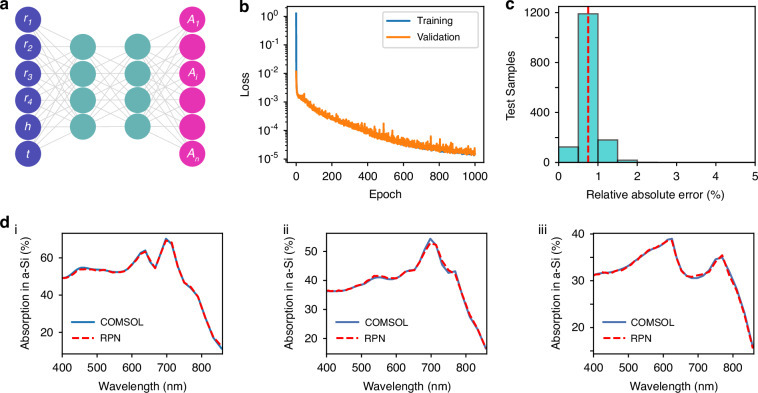
Proposed RPN for the design space of spectral response mapping. **a** Schematic of the RPN. **b** The decrease of training and validation loss during RPN training. **c** Statistical distribution of relative spectral error when applying the trained RPN on the test dataset, where the red dashed line shows the mean error. **d** Representative examples of predicted absorption spectra, where the solid blue and the dashed red curves indicate the target and RPN predicted results, respectively. The corresponding design parameters for (i–iii) are provided in the SI

In the forward modeling, one calculates the optical responses of a physical system, and there exists a one-to-one mapping between the physical design space and the associated spectral response, as illustrated in Fig. 2a. The employment of RPN replaces the conventional numerical simulations with a surrogate neural network model, predicting the response in fractions of milliseconds from the design parameters. Once trained, the neural network is typically orders of magnitude faster than the conventional forward modeling. The architecture of our RPN is 6−300−200−200−200−50−41, where 6 and 41 are, respectively, the number of inputs and outputs, and the remaining ones are the number of neurons in hidden layers. This setting of hyperparameters regarding neural network architecture is provided in the Supplementary Information (SI). The training process of the RPN is performed with mean squared error (MSE) loss function defined as ${\mathscr{L}}=\frac{1}{N}\sum _{i}{{||}{R}_{i}-{\hat{R}}_{i}{||}}^{2}$ where *R*_*i*_ and ${\hat{R}}_{i}$ are the actual and the predicted absorption response, respectively. As performing a regression problem, RPN predicts the spectrum based on given geometrical parameters. MSE is a natural choice for the loss function in this context because it penalizes larger prediction errors by squaring them. This characteristic of MSE encourages the model to reduce significant deviations, thereby focusing on minimizing substantial errors more aggressively. In Fig. 2b, the loss function drops sharply during training and performs consistently on validation, indicating effective learning of complex patterns for predicting the absorption spectrum from given arbitrary design parameters, without explicitly solving it.

As a measure of the accuracy of the trained RPN, we calculate the relative absolute spectral error over test datasets as: $e={\sum }_{i}\left|{R}_{i}-{\hat{R}}_{i}\right|/{R}_{i}$ where *R*_*i*_ and ${\hat{R}}_{i}$ represent the target and the predicted absorption responses. As shown in Fig. 2c, a comparison of COMSOL simulations and RPN predictions indicates that the network exhibits an accuracy of over 99% for predicting the absorption spectra. The simulated and predicted absorption spectra coincide as shown in Fig. 2d(i)–(iii).

### Design predicting network

The importance of using the DPN consists mainly in addressing the inverse design problem, which involves finding the optimal parameter for a desired response. This inverse design is associated with a unique set of challenges compared to the forward modeling, primarily since a simple response can be achieved through multiple distinct physical configurations, resulting in a many-to-one mapping.

**Fig. 3:**
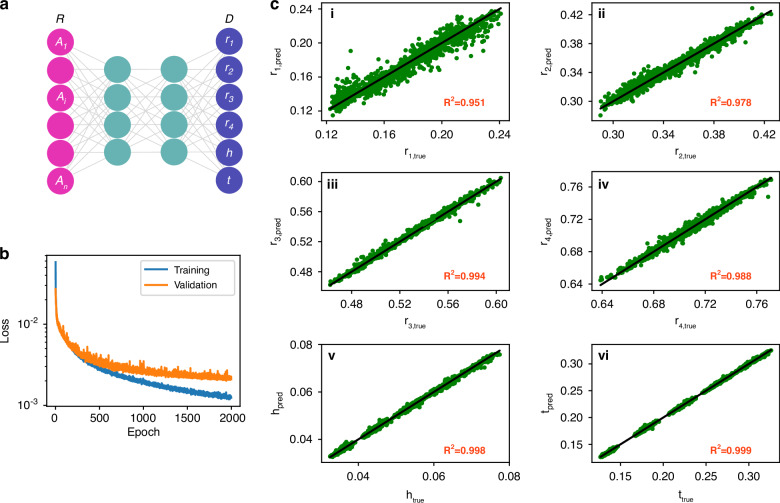
Proposed DPN for design parameters from the given absorption spectrum of the meta-absorber. **a** Schematic of the DPN. **b** The decrease of training and validation loss during DPN training. **c** The regression plots of normalized design space where the solid black line represents the best fit and green dots show the predicted parameters. The regression coefficients verify the effectiveness of the trained network as reported in (i)–(vi)

The non-uniqueness of the solution space has been reduced by applying a variety of regularization methods^54^, and deep learning strategies^55–60^ in the inverse design process, resulting in an approximate, well-defined solution. Using *L*_1_-norm or *L*_2_-norm on the data and regularization terms provides better robustness against sharp transitions and outliers for inverse problems^61^. The designed architecture of DPN is 41−400−300−300−200−200–50–6, where 41 and 6 are, respectively, the number of input and output, and others are the number of neurons in hidden layers. The hyperparameters are provided in the SI. To accelerate the training convergence, the design parameters are normalized using the *L*_2_-norm, mapping each sample to a unit-circle^62^. The loss function for training DPN is defined by the mean absolute error (MAE), ${\mathscr{L}}=\frac{1}{N}\sum _{i}|{\mathscr{D}_{i}}-{\hat{\mathscr{D}}}_{i}|$, where ${\mathscr{D}}_{i}$ and ${\hat{\mathscr{D}}}_{i}$ are the actual and the predicted design parameters, respectively. Using MAE rather than MSE as the loss function in DPN is because MAE offers robustness to outliers and noise in the data and treats all errors linearly and equally, which is crucial in inverse modeling. Figure 3b shows the decrease in training and validation loss, indicating a valid learning process of DPN. The training stopped at 2000 epochs to prevent overfitting when the validation loss began to decrease slowly. The trained DPN is evaluated on the testing set, by comparing the predicted design parameters with their simulation values. Figure 3c shows each of the six normalized design parameters against the predicted values output by the DPN for the testing data. The regression coefficients *R*^2^ approaching unity confirm a high degree of prediction accuracy of the trained network, as can be seen in Fig. 3c. The comparison of DPN performance with and without *L*_2_-normalization is presented in the SI (Fig. S6).

**Fig. 4:**
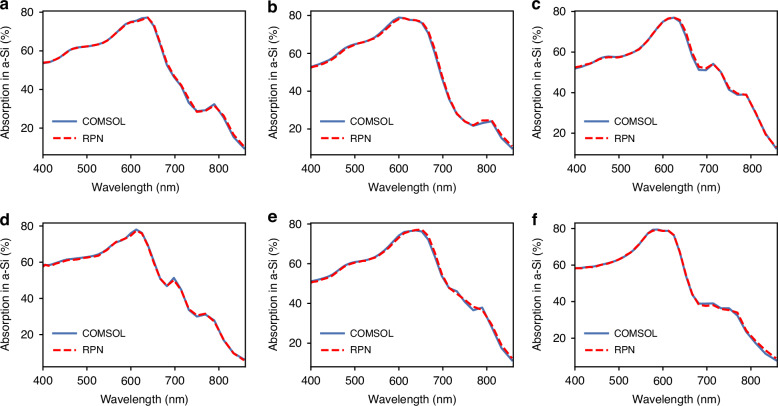
Inversely predicted design parameters from given absorption spectrum of the meta-absorber. **a**–**f** Solid line shows the target spectra computed from the COMSOL and the dashed line shows the RPN-predicted spectra from DPN-designed parameters. The DPN-designed parameters are provided in the SI

DPN designed parameters are further fed into the trained RPN to confirm the given absorption spectra. As shown in Fig. 4, the representative absorption spectra predicted by the RPN based on derived DPN design parameters coincide with the given absorption spectra (the predicted design parameters details are provided in the SI). The results indicate that light is absorbed strongly by the meta-absorber between 400 and 800 nm due to strong interactions between plasmonic and cavity modes. As the wavelength increases, the meta-absorber’s absorption capability decreases. In the region with wavelengths above 800 nm, the absorption rate is below 20% due to the poor intrinsic absorption of the a-Si.

### Broadband enhancement of light absorption

The designed RPN and DPN architectures are further exploited to analyze the performance of the proposed meta-absorber. In particular, we study the enhancement of the optical absorption in a spectrally dense region of air mass 1.5 solar irradiance *S*(*λ*). To quantify the performance of the device, the optical absorption of the a-Si active layer is weighed by the spectral solar irradiance, *S*(*λ*) and then integrated along the preferred wavelength range. Assuming that all charge carriers generated by photons contribute to the current generation in the active region, we can compute the photocurrent density defined as $J=\tfrac{q}{{hc}}{\int }_{{\lambda }_{1}}^{{\lambda }_{2}}\lambda {A}(\lambda )S\left(\lambda \right){\rm {d}}\lambda$ where *A* is the absorption spectrum, *h* is Plank’s constant, *λ* and *c* are the incident wavelength and speed of light in free space, respectively. *S*(*λ*) with a range from 400 to 860 nm is chosen based on the optical response of the a-Si. To measure the enhancement, we calculate the current with (*J*_w_), and without (*J*_wo_) nanorings structure inside the a-Si layer and compute the current enhancement *J*_enh_ defined as: *J*_enh_ =[(*J*_w_−*J*_wo_)/*J*_wo_] × 100%. The current enhancement strongly depends on the design parameters of the proposed meta-absorber.

**Fig. 5:**
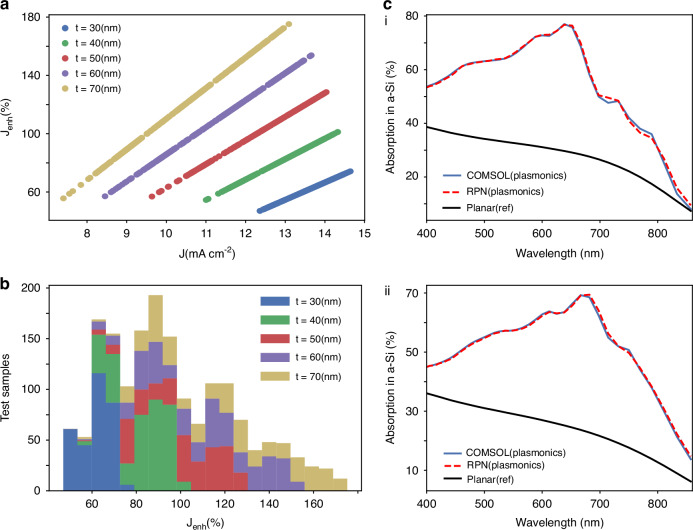
Multiple device performance analysis of designed meta-absorber. **a** Current density and current enhancement are plotted over the test data where different colors show how the current enhancement is affected by the spacer layer thickness. **b** The statistical distribution of enhancement current as a function of different spacer layer thicknesses is shown for the test data, indicating the enhancement in the range of 60–180% owing to the nanostructures. **c** Representative examples of enhanced broadband absorption where the solid blue line shows the computed spectra from the COMSOL, dashed red line shows the spectra predicted from RPN, and black solid line shows the absorption in reference structure without metallic rings. (i) *J* = 14.25 mA cm^−2^, *J*_enh_ = 100% for *D* = [29.44, 84.63, 110.41, 165.13, 13.83, 39.96] nm and (ii) *J* = 13.68 mA cm^−2^, *J*_enh_ = 123% for *D* = [35.45, 89.7, 120.32, 165.09, 11.98, 49.54] nm

Figure 5 shows the current density and the associated enhancement derived from the RPN predicted spectra over testing data. The thickness of the spacer layer allows the cavity modes to be tuned to match the plasmonic resonances for broadband absorption. Increasing the spacer layer thickness results in a larger enhancement, yet, it comes at the expense of small current density. Therefore, we need to choose the optimal parameters to achieve the desired current density with large enhancement. We compute the current enhancement for different sets of spacer layer thickness and present the corresponding statistical distribution as a guide to select the design space for enhanced performance (see Fig. 5b). Additionally, we provide the absorption spectra for optimal performance parameters and compare them with those of the reference structure to demonstrate the efficiency of our design in absorbing light in the ultra-thin silicon layer. Figure 5c illustrates RPN-predicted spectra using the DPN-derived design parameters that agree well with the COMSOL simulation results.

**Fig. 6:**
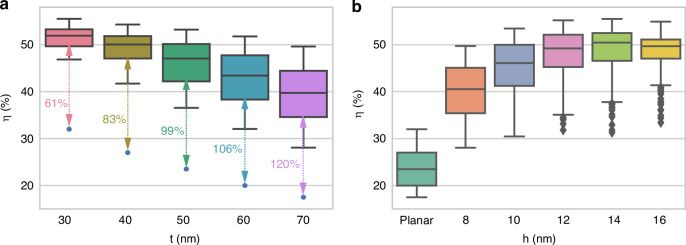
Multiple device statistical analysis of the designed meta-absorber. **a** The statistical distribution of efficiency of absorber for the testing data sets with different spacer layer and **b** double-ring nano structure thicknesses. The blue dots show the efficiency of the corresponding planar device. The results indicate efficiency over 50% is achieved with optimal design parameters of meta-absorber

We expect that the broadband absorption achieved by our design may benefit the high-performance photovoltaic devices^15,24^. To determine the efficiency of the absorber for potential solar cell applications, we calculate the ratio between absorption multiplied by incident irradiance, *S*(*λ*) and the total incident irradiance at the given wavelength. We illuminate the structure with standard AM1.5 G solar irradiance and assume recombination losses are neglected. The integrated efficiency is $\eta ={\int }_{{\lambda }_{1}}^{{\lambda }_{2}}A\left(\lambda \right)S\left(\lambda \right){\rm {d}}\lambda /{\int }_{{\lambda }_{1}}^{{\lambda }_{2}}S\left(\lambda \right){\rm {d}}\lambda$. Figure 6 depicts the statistical performance of the efficiency of the meta-absorber calculated from the RPN-predicted spectra. In Fig. 6a, the blue dots depict the efficiency of the planar device and the corresponding percentage increase. In comparison to the planar device, their multiple averages show positive increases when the spacer layer is thickened, similar to the enhancement shown in Fig. 5. Nevertheless, the maximum efficiency increases as the nanoring thickness increases, and then decreases, indicating that 50% efficiency can be achieved with a flexible thickness of between 10 and 14 nm.

### Experimental demonstration

**Fig. 7:**
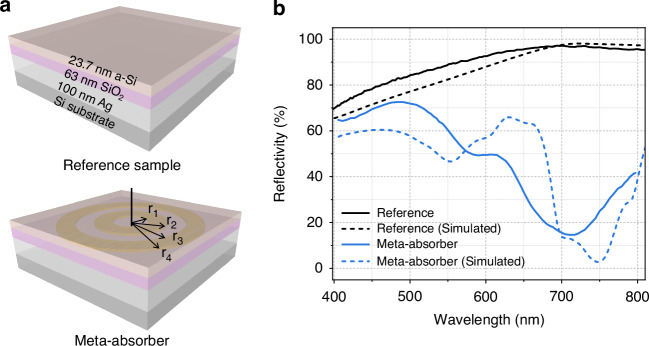
Schematic and reflection spectra of the studied structures. **a** The schematic of reference and meta-absorber containing silver nanoring array. The thickness of each layer is labeled. **b** The simulated and experimental reflection spectra of reference and meta-absorber

**Fig. 8:**
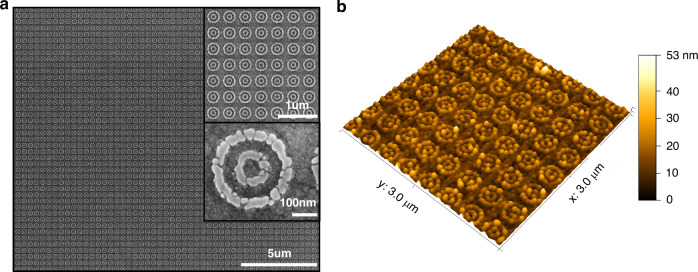
Morphology of the silver nanoring array. **a** The SEM micrograph of silver nanoring arrays with the magnification of ×16,000, ×50,000, and ×700,000, respectively. **b** The 3D AFM image shows a clear silver nanoring array

To validate our design, we fabricate a meta-absorber structure that exhibits a broadband enhancement effect. The fabrication process involves depositing various layers onto a silicon substrate. As depicted in Fig. 7a, the meta-absorber consists of a 100-nm-thick silver metallic layer, a 63-nm-thick SiO_2_ dielectric layer, the silver double nanoring array, and a 23.7-nm-thick a-Si thin film. All the fabrication details are given in the “Methods” section. The nanoring was fabricated by using electron beam lithography because its size dimensions are in the deep subwavelength scale. Figure 8a shows a scanning electron micrograph (SEM) of the silver nanoring arrays after the lift-off process (before deposition of the a-Si). The silver nanoring is characterized by inner and outer ring radii of *r*_1_ = 40 nm, *r*_2_ = 80 nm, *r*_3_ = 120 nm, and *r*_4_ = 160 nm, respectively; and all the structures are arranged in a square array with a 400-nm pitch. These microscopic images in Fig. 8a with different magnifications exhibit excellently arranged silver nanorings with high quality in their shapes. A three-dimensional atomic force microscopy (AFM) image of silver nanoring array is shown in Fig. 8b. The morphology of double nanoring is clearly discerned, while the uniformity of the nanoring’s thickness is often compromised, given that the material has just begun to nucleate for the ultrathin nanofilms. In height profiles extracted from AFM, the cross-sectional circular segment shape is observed, and the vertex has the height fluctuation. By extracting the height profile of several nanorings, we choose 12 nm as the effect thickness of silver nanorings (details are provided in the SI). These feature sizes are also adopted in the simulation.

For comparison, the control sample without the silver nanoring array, but using the same process, has also been fabricated for reference and is illustrated in Fig. 7a. Experimental and simulated reflection spectra for the reference and meta-absorber are displayed in Fig. 7b. The measurement details are provided in the Methods section. A comparison with the experimental spectra of the reference and meta-absorber reveals that the most prominent dips from 630 to 800 nm correspond to the broadband absorption of the silver nanoring array. The simulated spectra also clearly show that the silver nanoring array enhances the absorption within broadband from 680 to 800 nm, which is basically consistent with the experimental spectra. These results demonstrate that the silver nanoring array performs as a meta-absorber at the reflection mode and provides a broadband enhancement effect. A comparison with the previous metasurface and/or plasmonic absorbers is shown in the SI.

## Discussion

Our research presents an approach for enhancing broadband absorption in ultra-thin silicon films through the utilization of machine learning in the design process. The proposed plasmonic meta-absorber incorporates 2D periodic arrays of plasmonic nanorings. The absorber’s absorption capability is attributed to the resonant absorption of the cavity and near-field enhancement of silver nanorings structures in the a-Si active region. By carefully adjusting the nanorings’ geometric parameters and the thickness of the spacer dielectric layer, we can precisely control the absorption enhancement in the ultra-thin silicon layer. Using a deep learning approach, we design RPN and DPN and observe significant improvements in the absorber’s performance: an optimum photocurrent enhancement of over 100% is achieved in the absorber compared to a reference structure without plasmonic nanostructures. Our research not only provides insights into the design and optimization of plasmonic meta-absorbers but also demonstrates the efficacy of deep learning techniques in enhancing the performance of such devices. The use of a data-driven approach, although requires a big one-time cost in data generation, can explore high-dimensional design spaces more efficiently and precisely compared with the traditional methods, such as built-in methods in COMSOL (details discussed in the SI). In this study, we chose rings, the highly symmetric and regular geometry, as the nanostructure to demonstrate our approach. We would like to point out that irregular structures may bring more degrees of freedom and may offer potential advantages for optimizing absorption properties, which, in principle, can also be addressed by our design strategy, while the experimental implementation may become more challenging. Nevertheless, the ability to precisely control and tailor the optical properties of the absorber holds great promise for the advancement of optoelectronics, paving the way for the development of highly efficient and customizable spectral selective absorbers.

## Methods

### Fabrication

The fabrication started with 4’ wafers of Si(111) that were cut into 1 cm × 1 cm squares with a Ytterbium fiber laser. The resulting substrates were thoroughly cleaned in an ultrasonic bath of acetone, then rinsed in isopropanol and deionized water, and finally blown dry with nitrogen. On the cleaned substrate, a 1-nm-thick Ge layer (evaporation rate: 0.1 Å s^−1^) as an adhesion layer was first electron beam (e-beam) evaporated. Then, the reflective layer, a 100-nm-thick silver (evaporation rate: 10 Å s^−1^), was deposited in the same batch. Subsequently, a thin SiO_2_ dielectric layer was deposited by magnetron sputtering at room temperature (RT). Its thickness was confirmed by Filmetrics F2-RT. The sample was then ready for the fabrication of the double-nanoring arrays using e-beam lithography and the lift-off procedure. Firstly, the nanoring arrays were prepared by spin-coating a positive resist onto the substrate at 6000 rpm for 60 s. The resist used for this step was a solution of ARP6200.09 diluted in anisole, its thickness after a hot-plate bake at 150 °C for 3 min was about 75 nm. The resist was exposed by a 100 kV electron beam inside the JEOL Electron Beam Lithography System without using the proximity effect correction function. The exposition dose was adjusted according to the resist thickness and pattern. After developing the exposed resist (with the AR600-546 developer), a 2-nm-thick ITO adhesive layer and 10-nm-thick silver layer were e-beam evaporated (evaporation rate: 0.1 Å s^−1^ for both metals. Finally, lift-off was carried out by dipping the samples into the resist remover solvent (AR600-71). The final step, after the fabrication of the nanoring arrays, was the deposition of the a-Si thin film, which was performed with the plasma-enhanced chemical vapor deposition (PECVD) process. Here, the a-Si thickness is confirmed by the Filmetrics F2-RT.

### Structural, optic, electron, and atomic force microscopy characterization

Scanning electron microscopy images were recorded by an eSEM-FEI Quattro-S instrument operating at an accelerating voltage of 5 kV. The reflectance spectra measurements were performed with the Nanospec 6100 reflectometer coupled with an optical microscope. The structures were illuminated from the a-Si side with a microscope in the normal direction. The data were normalized by the reflectivity of a substrate without nanoring arrays and the a-Si layer. Atomic force microscopy images were acquired in the air under ambient conditions using a Dimension Icon SPM scanner system. Measurements were made in tapping mode using an RTESPA-300 (Bruker) tip with a spring constant *k* = 40 N m^−1^ and a resonance frequency of 300 kHz. The thin film thickness and optical constants (*n, k*) were characterized using Filmetrics F2-RT with LS-DT2 light source. The thickness of the e-beam positive resist was measured by the DektakXT stylus surface profilometer.

## Supplementary information

Supplemental information

## Data Availability

The source data are available from the corresponding authors upon reasonable request.
