# Supplementary material for: Machine learning assisted plasmonic metascreen for enhanced broadband absorption in ultra-thin silicon films
Source: Light Sci Appl. 2025 Jan 9;14:42. doi: 10.1038/s41377-024-01723-8 (PMC11711677; doi:10.1038/s41377-024-01723-8)
Supplement: Supplementary file 1 — Supplemental information [file 41377_2024_1723_MOESM1_ESM.docx]

**Supplementary information for**

**“Machine learning assisted plasmonic metascreen for enhanced broadband absorption in ultra-thin silicon films”**

Waqas W. Ahmed^1†^, Haicheng Cao^1†^, Changqing Xu^1†^, Mohamed Farhat^1^, Muhammad Amin^2^, Xiaohang Li^1,3^, Xiangliang Zhang^1,4§^, and Ying Wu^1,3*^

^1^Division of Computer, Electrical and Mathematical Sciences and Engineering, King Abdullah University of Science and Technology (KAUST), Thuwal, 23955-6900, Saudi Arabia

^2^College of Engineering, Taibah University, Madinah 42353, Saudi Arabia

^3^ Division of Physical Science and Engineering, King Abdullah University of Science and Technology (KAUST), Thuwal, 23955-6900, Saudi Arabia

^4^ Department of Computer Science and Engineering, University of Notre Dame, Notre Dame, IN 46556, United States of America

Emails: [^*^ying.wu@kaust.edu.sa](file:///C:\Users\AHMEDWW\Documents\ML\*ying.wu@kaust.edu.sa), **^§^**xzhang33@nd.edu

**^†^**Equally contributed as the first author.

**S1. Training of response predicting network (RPN) & design prediction network (DPN)**

RPN and DPN models are built and tested using Keras and Tensorflow - open-source machine learning frameworks based on Python. In order to train the networks, we apply the Adam algorithm for stochastic gradient descent. The learning rate is initially set to 0.0002 and the batch size is 50. ReLU activation function is employed for fully connected layers during the training process. More examples for the trained RPN and DPN are provided in the Fig.S1 and Fig.S2, respectively.

**
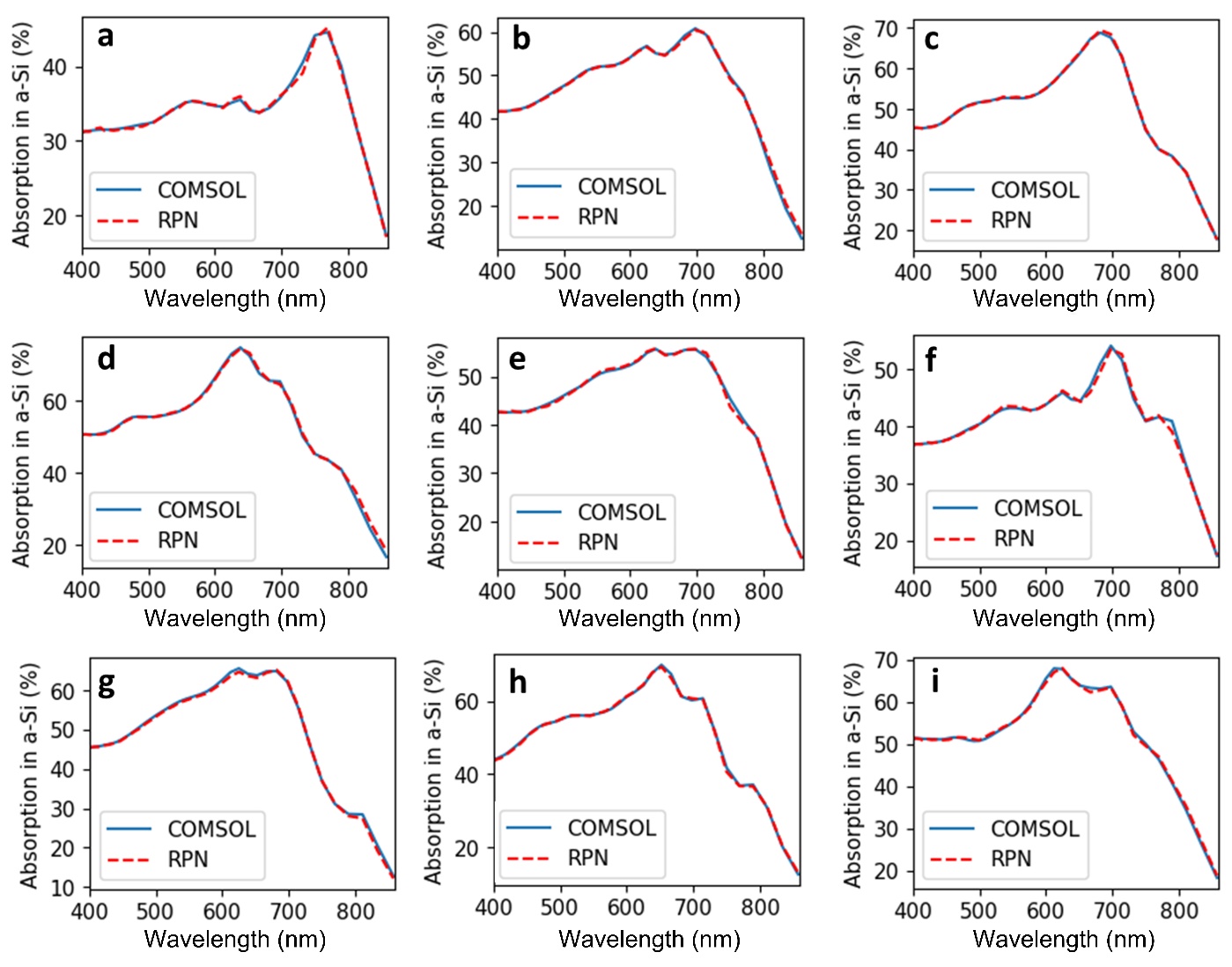
**

Fig. S1 Predicted response by RPN on given design parameters. The solid blue and dotted red line represent the absorption spectra from COMSOL and ML method (RPN), respectively. The neural architecture is the same as the one in the main text. The design parameters of predicted spectra are provided in Table S1.

**
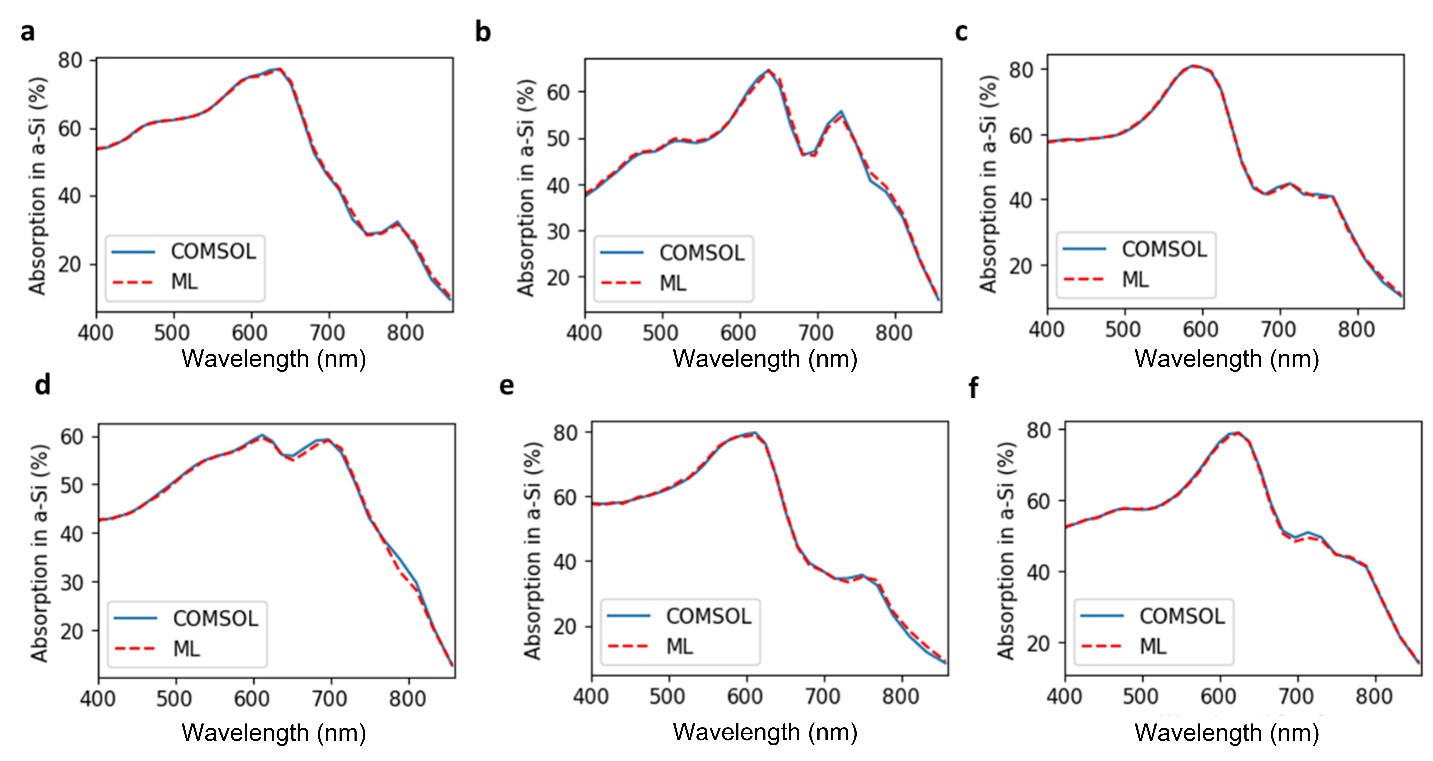
**

Fig. S2 Predicted design parameters by DPN given spectra response. The solid blue and dotted red line represent the absorption spectra from COMSOL and ML method (DPN), respectively. The neural architecture is the same as the one in the main text. The predicted design parameters are provided in Table S2.

Table S1: Corresponding design parameters for the predicted responses using RPN.

| **Figure** | $r_{1}$(nm) | $r_{2}$(nm) | $r_{3}$(nm) | $r_{4}$(nm) | $t$(nm) | $h$(nm) |
| --- | --- | --- | --- | --- | --- | --- |
| Fig.2d(i) | 50 | 90 | 110 | 150 | 16 | 50 |
| Fig.2d(ii) | 35 | 70 | 130 | 160 | 10 | 60 |
| Fig.2d(iii) | 40 | 85 | 110 | 150 | 8 | 70 |
| Fig. S1a | 45 | 75 | 130 | 170 | 8 | 70 |
| Fig. S1b | 35 | 80 | 120 | 165 | 10 | 50 |
| Fig. S1c | 50 | 75 | 115 | 150 | 12 | 40 |
| Fig. S1d | 50 | 80 | 120 | 150 | 14 | 30 |
| Fig. S1e | 30 | 75 | 130 | 170 | 8 | 40 |
| Fig. S1f | 40 | 85 | 130 | 160 | 10 | 60 |
| Fig. S1g | 30 | 70 | 110 | 165 | 10 | 40 |
| Fig. S1h | 40 | 80 | 110 | 165 | 14 | 60 |
| Fig. S1i | 35 | 70 | 125 | 150 | 16 | 30 |

Table S2: Corresponding predicted design parameters for the given responses using DPN.

| **Figure** | $r_{1}$(nm) | $r_{2}$(nm) | $r_{3}$(nm) | $r_{4}$(nm) | $t$(nm) | $h$(nm) |
| --- | --- | --- | --- | --- | --- | --- |
| Fig.4a | 45.01 | 80.16 | 109.76 | 170.03 | 14.05 | 40.41 |
| Fig.4b | 34.24 | 74.99 | 110.08 | 169.88 | 11.83 | 29.95 |
| Fig.4c | 44.46 | 89.27 | 130.41 | 170.31 | 14.18 | 40.03 |
| Fig. 4d | 45.17 | 90.99 | 110.3 | 69.23 | 15.93 | 39.7 |
| Fig. 4e | 49.72 | 89.89 | 125.45 | 164.95 | 11.85 | 29.95 |
| Fig. 4f | 34.15 | 74.81 | 114.95 | 170.29 | 16 | 29.92 |
| Fig. S2a | 45.01 | 80.16 | 109.76 | 170.03 | 14.05 | 40.41 |
| Fig. S2b | 39.96 | 89.71 | 129.71 | 170.37 | 14.11 | 69.99 |
| Fig. S2c | 34.65 | 79.95 | 124.82 | 170.19 | 16.04 | 29.98 |
| Fig. S2d | 34.77 | 84.83 | 109.95 | 165.1 | 9.91 | 49.72 |
| Fig. S2e | 43.7 | 74.85 | 115.15 | 170.26 | 15.97 | 29.89 |
| Fig. S2f | 35.01 | 74.34 | 130.24 | 165.27 | 14.05 | 29.95 |

**S2. Examples of Broadband Absorption enhancement**

Fig.S3 shows additional examples of broadband absorption enhancement using planar devices without plasmonic rings. The design parameters and corresponding performance characteristics are provided in Table S3.

**
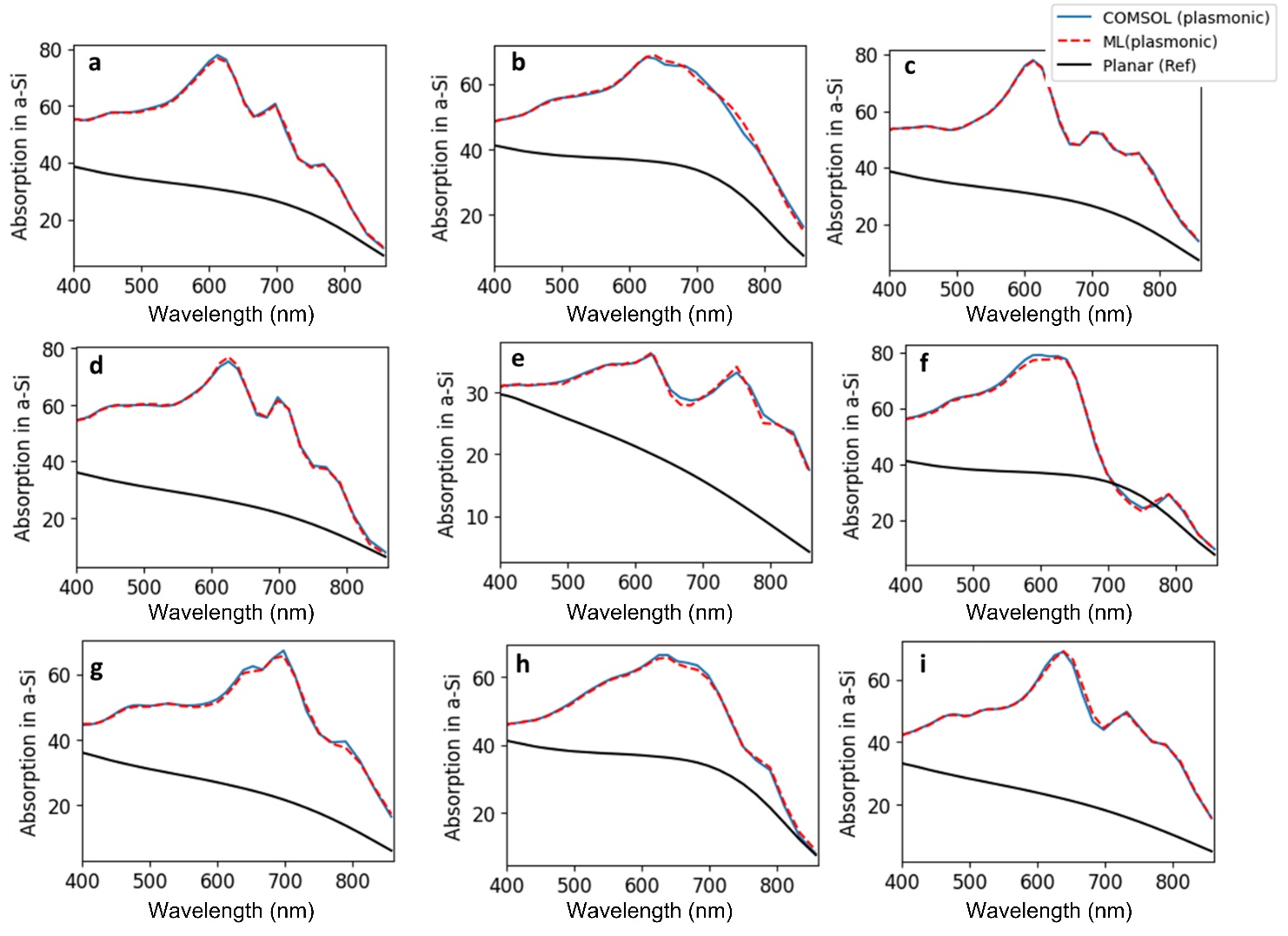
**

Fig. S3 Results of the trained design predicted network for the given spectra. The solid blue and dotted red line represent the absorption spectra from COMSOL and ML method (DPN), respectively. The solid black line is plotted to show the reference spectra for the planar device. The neural architecture is the same as the one in the main text. The design parameters of predicted spectra are provided in Table S1.

Table: S3 Corresponding predicted design parameters for the given responses using DPN and performance characteristics

| **Figure** | $r_{1}$(nm) | $r_{2}$(nm) | $r_{3}$(nm) | $r_{4}$(nm) | $t$(nm) | $h$(nm) | $J$  (Am cm^-2^) | $J_{enh}$  $(\%)$ | $\eta$ |
| --- | --- | --- | --- | --- | --- | --- | --- | --- | --- |
| Fig.S3a | 50.04 | 90.4 | 120.04 | 164.91 | 15.96 | 40.14 | 13.78 | 94 | 0.52 |
| Fig.S3b | 39.66 | 84.25 | 125.23 | 150.26 | 11.93 | 29.74 | 14.03 | 67 | 0.53 |
| Fig.S3c | 30.62 | 70.15 | 14.93 | 165.01 | 15.98 | 39.98 | 13.56 | 90 | 0.51 |
| Fig. S3d | 40.7 | 89.91 | 110.02 | 165.12 | 16.09 | 49.42 | 13.69 | 123 | 0.52 |
| Fig. S3e | 35.09 | 84.99 | 124.82 | 149.86 | 7.93 | 70.7 | 8.08 | 70 | 0.31 |
| Fig. S3f | 43.43 | 76.27 | 109.45 | 169.17 | 13.96 | 29.7 | 13.46 | 60 | 0.51 |
| Fig. S3g | 46.78 | 72.25 | 109.94 | 148.67 | 13.66 | 49.41 | 12.87 | 110 | 0.49 |
| Fig. S3h | 33.66 | 87.82 | 124.61 | 163.24 | 7.83 | 29.4 | 12.94 | 54 | 0.49 |
| Fig. S3i | 29.86 | 74.97 | 130.12 | 170.33 | 13.95 | 60.13 | 12.57 | 134 | 0.48 |

**S3. Simulations and the field distribution**

The simulations in our manuscript are calculated by using the Electromagnetic waves module in COMSOL MULTIPHYSICS. We run the simulations on a High-End Scientific Workstation (Supermicro SYS-7049A-T, with 2 Intel Xeon Gold 6130 processor and 1 TB memories). Fig. S1(a) shows a schematic of a quarter of the simulated area. Silicon, silver rings, and silicon dioxide are sequentially deposited on a silver substrate. Their permittivities and permeabilities are arrays obtained from experiments. Light shines such a system vertically from the air. Fig. S1(b-d) are top view of the electric field for the case of maximum absorption at a frequency around 400 THz, showing a resonant mode in the silver ring. Fig. 2 is the $E_{x}$ field distributions near the sliver rings in the (a) *x-z* slice and (b) *y-z* slice of the simulation results at the frequency of 400 THz.


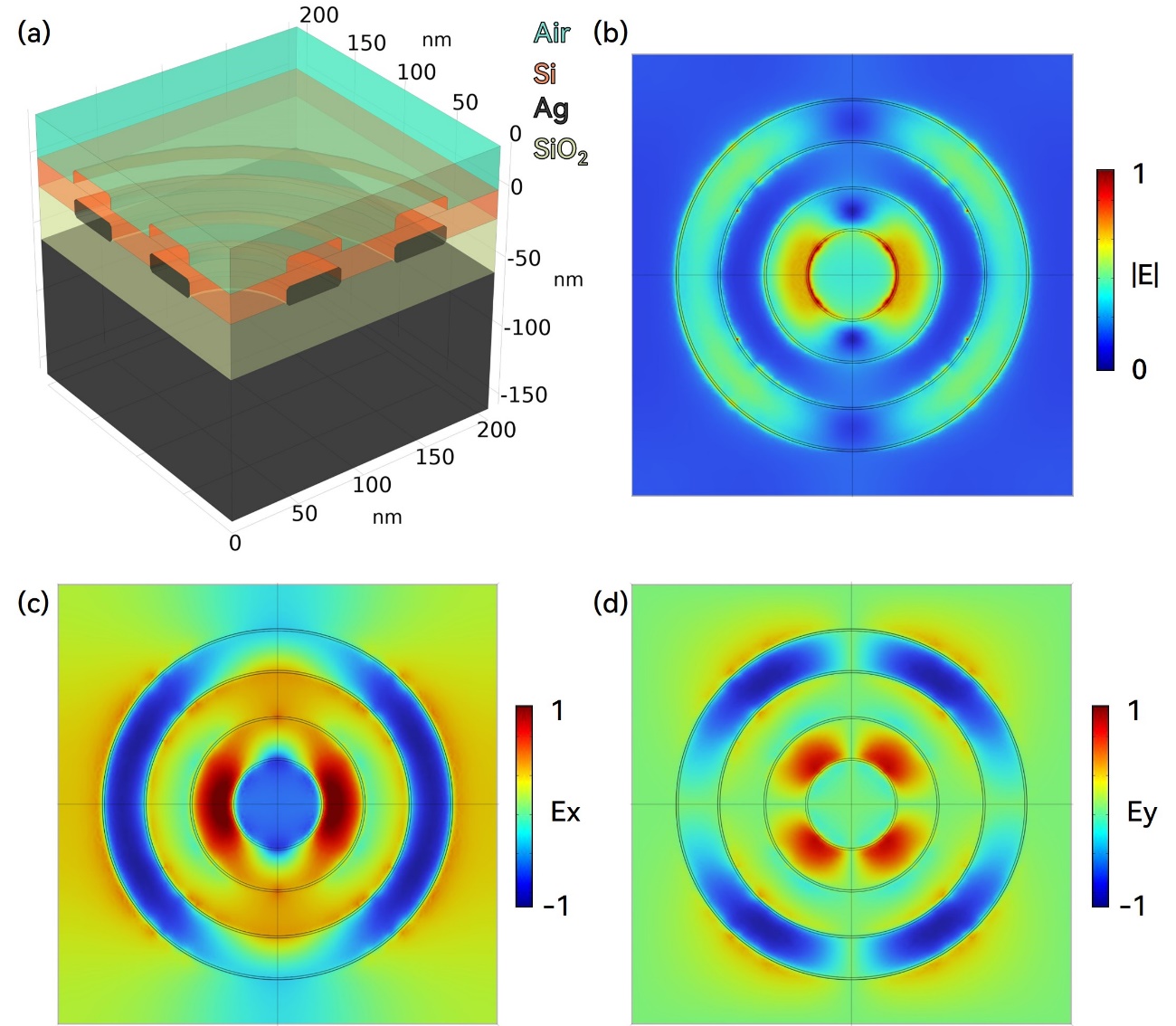


Fig. S4. (a) The sketch of the simulated region in COMSOL. (b-d) Top view of the (b) amplitude, (c) *x* component and (d) *y* component of electric field at a frequency 400 THz.


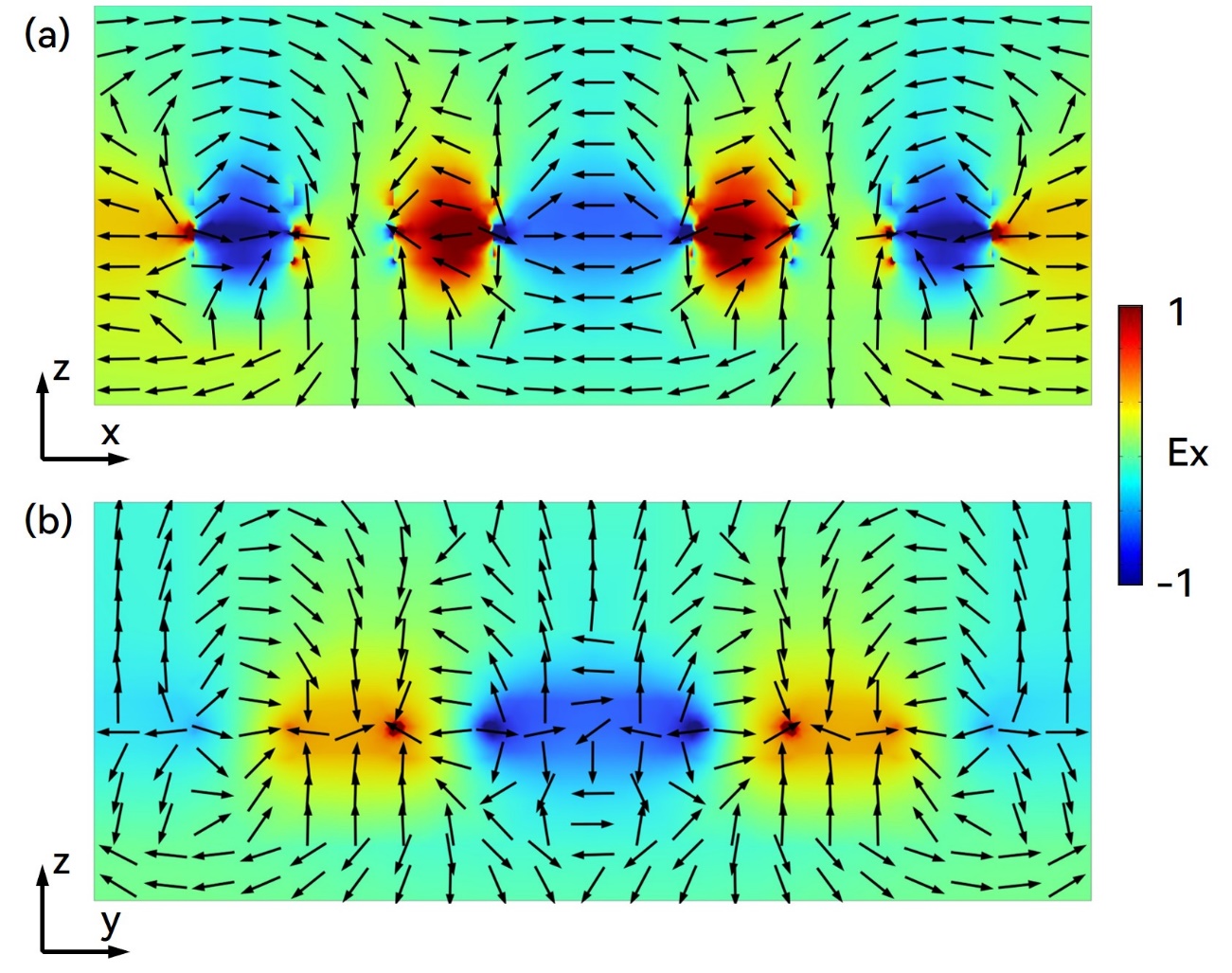


Fig. S5. $E_{x}$ field distributions near the sliver rings in the (a) *x-z* slice and (b) *y-z* slice of the simulation results at the frequency of 400 THz.

**S4. The AFM characterization and height profile of the Ag nanoring array**

Fig. S6 (a) shows AFM view of the nanoring array and the line’s positions to extract the height profile. The height profile of line 1 and line 2 were discipled in Fig. S6(b). From the height data in two lines, its effective height could be estimated to be 14nm. So, the Ag thickness could be 12 nm considering 2 nm ITO adhesive layer.


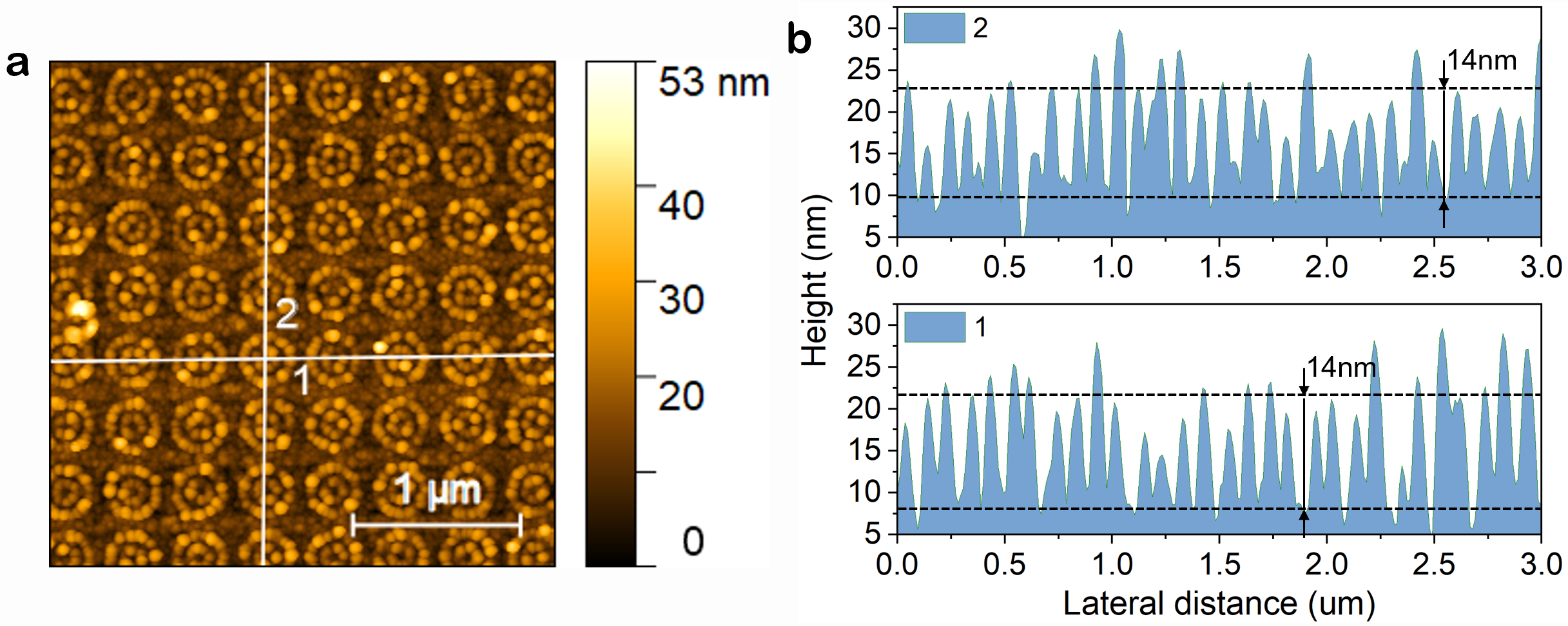


Fig.S6 (a) AFM view of the nanoring array, and the line’s positions to extract the height profile; (b) the height profile of line 1 and line 2, respectively.

**S5. Comparison of DPN performance with and without L_2_ normalization.**

In DPN design, the $L_{2}$ normalization of data improves the predictions of DPN significantly; as shown in Fig. S6. The regression coefficients for the ring radii $r_{1}$, $r_{2}$, $r_{3}$ and $r_{4}$ result in improvements of 31.2 %, 26 %, 7.6 %, 2.3 %, respectively as compared to training DPN without normalization.


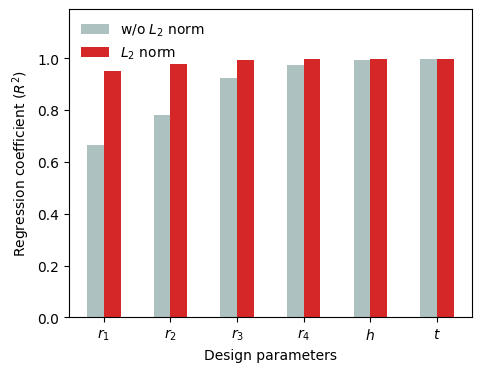


Fig.S6 Effect of regularization of data on the DPN prediction performance.

**S6. Comparison of the reported work and the previously studied absorbers at similar frequency ranges**

Table S4. Absorbers integrated with nano structures.

| **Study** | **Design** | **Material & Thickness** | **Wavelength**  **(nm)** | **Absorption**  **Enhancement (%)** | **Photocurrent enhancement(%)** |
| --- | --- | --- | --- | --- | --- |
| This work | Ag double Nanorings | a-Si, 20 nm | 400-850 | > 125 | >125 |
| Pala et. al. (2009)^1^ | 2D Ag-metallic strips | a-Si, 50 nm | 300-1100 |  | 43 |
| Guilatt et. al. (2010)^2^ | Silver nano shell | Si, 50 nm | 300-1000 | 18 |  |
| Wei et. al. (2010)^3^ | Silver nano-grating | a-Si, 100 nm | 270-950 | 30 |  |
| Sreekanth et. al. (2011)^4^ | Silver nanoparticles | Si, 200 nm | 400-800 | 10.2 |  |
| Ho et. al. (2012)^5^ | Multilayer Gold nanoparticles | a-Si | 800 |  | 9.8 |
| Zhu et. al. (2013)^6^ | Plasmonic fractal | Si, 50 nm | 450 |  | 117 |
| Kazi et. al. (2014)^7^ | Gold nanoparticles | a-Si, 20 nm | 300-800 |  | 8.46 |
| Raja et. al. (2016)^8^ | Core-shell | c-Si, 300 nm | 400-1100 |  | 21 |
| Aminfarad et. al. (2019)^9^ | Silver nanospheres with silica shells | a/c-Si, 100 nm | 400-1100 | 88 |  |
| Hongen et. al. (2020)^10^ | SiO2 nanoparticles + Ag Hemisphere | a-Si, 100 nm | 400-800 |  | 21 |
| Fazal et. al. (2020)^11^ | Bi-metallic nanograting | a-Si, 200 nm | 300-1200 | 40 |  |
| Hongen et. al. (2021)^12^ | Double-layer antireflection coating and Ag hemispheres | a-Si, 100 nm | 350-800 |  | 39 |
| Li et. al. (2022)^13^ | Silicon rods on silica | a-Si rods, 220 nm height | 736 | Undefined (No absorption without Si rods) | Undefined (No absorption without Si rods) |
| Nishida et. al. (2023)^14^ | Silicon rods on silica | a-Si rods, 79 nm height | 450 and 550 (two peaks) | Undefined (No absorption without Si rods) | Undefined (No absorption without Si rods) |

1. Pala, Ragip A., et. al. "Design of plasmonic thin-film solar cells with broadband absorption enhancements." Adv Mater. 21:3504-3509 (2009).
2. Guilatt, Oren, Boris Apter, and Uzi Efron."Light absorption enhancement in thin silicon film by embedded metallic nanoshells," Opt. Lett. 35, 1139-1141 (2010).
3. Wang, W., et. al. "Broadband light absorption enhancement in thin-film silicon solar cells." Nano letters, 10(6), 2012-2018 (2010).
4. Sreekanth, K. V., R. Sidharthan, and V. M. Murukeshan. "Gap modes assisted enhanced broadband light absorption in plasmonic thin film solar cell." J. Appl. Phys. 110, 033107 (2011).
5. Ho, Chung-I., et. al. "Plasmonic multilayer nanoparticles enhanced photocurrent in thin film hydrogenated amorphous silicon solar cells." Journal of Applied Physics J. Appl. Phys. 112, 023113 (2012).
6. Zhu, Li-Hao, et. al. "Broadband absorption and efficiency enhancement of an ultra-thin silicon solar cell with a plasmonic fractal." Opt. Express 21, A313-A323 (2013).
7. Islam, Kazi, et. al."Effect of gold nanoparticles size on light scattering for thin film amorphous-silicon solar cells." Solar Energy 103, 263-268 (2014).
8. Raja, W., Bozzola, A., Zilio, P. et al. Broadband absorption enhancement in plasmonic nanoshells-based ultrathin microcrystalline-Si solar cells. Sci Rep 6, 24539 (2016).
9. Aminfard, Sam, Richard K. Harrison, and Adela Ben-Yakar. "Enhanced optical absorption in ultrathin silicon films using embedded silica-coated silver nanoparticles." Optics Communications 430, 143-150 (2019).
10. Li, Hongen, et. al. "Theoretical investigation of broadband absorption enhancement in a-Si thin-film solar cell with nanoparticles." Sol. Energy Mater. Sol. Cells, 211, 110529 (2020).
11. Subhan, F.E., et. al. "Efficient broadband light absorption in thin-film a-Si solar cell based on double sided hybrid bi-metallic nanogratings."RSC Adv. 10, 11836–11842 (2020).
12. Li, Hongen, et. al. "Full‐spectrum absorption enhancement in a‐Si: H thin‐film solar cell with a composite light‐trapping structure." Solar RRL 5, 3, 2000524 (2021).
13. Li. Y., et. al. “Ultra-narrow band perfect absorbance induced by magnetic lattice resonances in dielectric dimer metamaterials.” Results in Physics 39, 105730 (2022).
14. Nishida K. et. al. “All-optical scattering control in an all-dielectric quasi-perfect absorbing Huygens’ metasurface.” Nanophotonics 12, 139–146 (2023).

**S7. Traditional optimization and advantage of using data-driven approach.**

In a data-driven approach, such as using neural networks for inverse design, require big one-time cost for generating data. However, compared with traditional approaches (e.g., built-in methods in COMSOL), the data-driven approach can explore high-dimensional design spaces more efficiently and precisely. This efficiency can lead to the discovery of novel designs or the optimization of existing ones with fewer computational resources and iterations. In addition, although there is an initial investment in generating training data, this upfront cost results in significant long-term savings in computational time and resources. Traditional methods often require extensive manual parameter tuning, repeated simulations, or iterative adjustments, all of which are time-consuming and computationally intensive. In contrast, a well-trained neural network accelerates the design process by learning the mapping from input parameters to desired outputs. For instance, a single COMSOL simulation takes about 2 minutes to solve a three-dimensional absorber structure in our work, a trained neural network, such as the RPN, can generate comparable results in a few milliseconds.

The results of a traditional model optimization are often local optimal and often vary with different initial conditions, while the results obtained by our method are global optimal and are relatively stable. For example, we used Nelder-Mead solver in COMSOL Optimization Module, with a target of maximizing the averaged absporption over the desired freqeuncy range as discussed in the manuscript. It took about 5 hours to get the results with different initial conditions. In Fig. S7, we selected to examples calculated in COMSOL. For the blue curve, the upper and lower bounds of the optimization space are set as ${30 nm\leq\text{ }r}_{1}\leq50 nm$, ${70 nm\leq\text{ }r}_{2}\leq90 nm$, ${110 nm\leq\text{ }r}_{3}\leq130 nm$, ${150 nm\leq\text{ }r}_{4}\leq170 nm$,$8 nm\leq\text{ }h\leq16 nm$ and $30 nm\leq\text{ }t\leq70 nm$. The optimized result is close to that shown in Fig. 5c in main text. For the red curve, the upper and lower bounds of the optimization space are set as ${20 nm\leq\text{ }r}_{1}\leq58 nm$, ${60 nm\leq\text{ }r}_{2}\leq98 nm$, ${100 nm\leq\text{ }r}_{3}\leq145 nm$, ${150 nm\leq\text{ }r}_{4}\leq190 nm$,$8 nm\leq\text{ }h\leq16 nm$ and $30 nm\leq\text{ }t\leq70 nm$. For both cases, the initial conditions are close to the middle of the upper and lower bounds. It is obvious that although the absorption at 550 nm ~ 650 nm is high, the overall absorption is compromized. This demonstrates the substantial efficiency gains achievable with data-driven approaches. For other algorithms such as Monte Carlo solver, the optimization in COMSOL does not get stuck in local minima, but the optimization is very slow. In addition, from the data-driven approach, once the deep learning network is trained, it can be used to predict the absorption with specific geometric parameters, and can also be used to predict or enhance the absorption for specified wavelength range, which is not possible with the built-in methods in COMSOL. In COMSOL, changes in the objective function or initial conditions require re-calculation, which is inefficient compared to our approach.


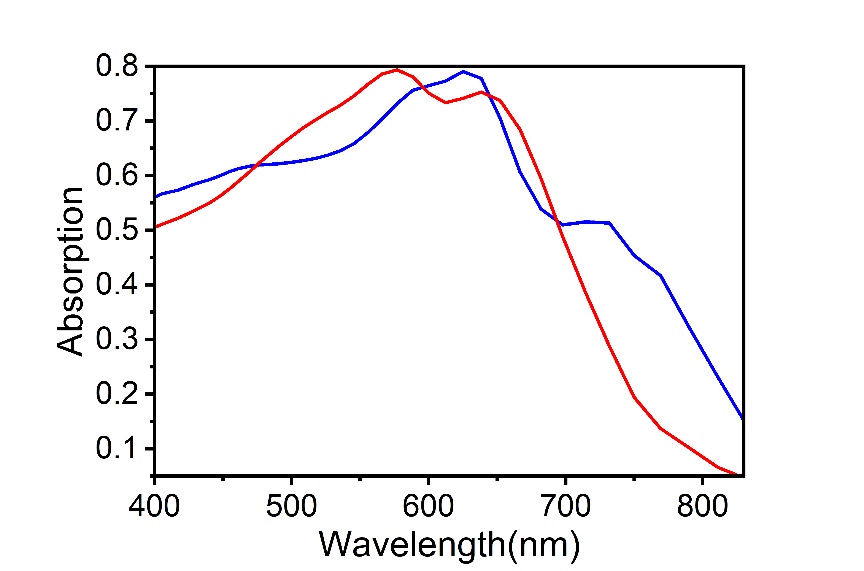


Fig. S7. Two optimized results from COMSOL Optimization Module with different initial conditions.
